# Medicines for headache before and during pregnancy: a retrospective cohort study (ATENA study)

**DOI:** 10.1007/s10072-020-04702-0

**Published:** 2020-09-23

**Authors:** Chiara Lupi, Andrea Negro, Elisabetta Gambassi, Tommaso Susini, Pierangelo Geppetti, Silvia Benemei

**Affiliations:** 1 Headache Centre, Careggi University Hospital, Florence, Italy; 2 Regional Referral Headache Centre, Sant’Andrea Hospital, Department of Clinical and Molecular Medicine, Sapienza University, Via di Grottarossa 1035-1039, 00189 Rome, Italy; 3 Maternal and Child Department, Careggi University Hospital, Department of Health Sciences, University of Florence, Florence, Italy; 4 Department of Health Sciences, University of Florence, Florence, Italy

**Keywords:** Drugs, Headache, Migraine, Cohort study, Pregnancy, Fetal risk

## Abstract

**Objective:**

To investigate headache treatment before and during pregnancy.

**Background:**

Most headaches in pregnancy are primary disorders. Headaches are likely to ameliorate during pregnancy, although they may also begin or worsen. Most headache medications should be avoided during pregnancy because of potential fetal risks. However, only scarce evidence on headache drug consumption during pregnancy is available.

**Design:**

ATENA was a retrospective, self-administered questionnaire-based, cohort study on women in either pregnancy or who have just delivered and reporting headache before and/or during pregnancy.

**Results:**

Out of 271 women in either pregnancy or who have just delivered, 100 (37%) reported headache before and/or during pregnancy and constituted our study sample. Before pregnancy, the attitude toward the use of symptomatic drugs was characterized by both a strong focus on their safety and the willingness to avoid possible dependence from them. Compared to the year before, pregnancy led to changes in behavior and therapeutic habits as shown by a higher proportion of patients looking for information about drugs (44/100 [44%] vs. 36/100 [36%]) and a lower proportion of those treating headache attacks (88/100 [88%] vs. 52/100 [52%]) and by a lower use of nonsteroidal anti-inflammatory drugs (68/100 [68%] vs. 5/100 [5%]) and a much higher use of paracetamol (33/100 [33%] vs. 95/100 [95%]).

**Conclusions:**

Pregnancy changes how women self-treat their headache, and leads to search for information regarding drug safety, mostly due to the perception of fetal risk of drugs. Healthcare providers have to be ready to face particular needs of pregnant women with headache.

**Electronic supplementary material:**

The online version of this article (10.1007/s10072-020-04702-0) contains supplementary material, which is available to authorized users.

## Background

Headache is a common symptom in women, affecting almost 20% of female general population as a primary disorder [1]. The occurrence of headache is influenced by female hormonal changes throughout the life cycle [2], and a large portion of pregnant women refers primary headaches, including tension-type headache and migraine [3]. Although the gestation, mainly during the last two trimesters, is usually associated with a decrease of attack frequency and severity in migraineurs [4, 5], 4–8% of patients report a worsening of symptoms and, in some subjects, migraine appears for the first time in the first trimester of pregnancy [6]. Several critical issues affect the management of headache, and particularly migraine, in pregnancy. On the one hand, migraine per se is a risk factor for gestational complications, such as hypertension, preeclampsia, and ischemic stroke [7], and it can lead to impaired nutritional intake, dehydration, sleep deprivation, high stress, and depression with associated adverse events on maternal and fetal well-being, when untreated or poorly managed [8]. On the other hand, the consumption of medications, with proven or unknown teratogenic potential, to treat [9, 10] or prevent [11–13] headache attacks can result in fetal malformations. Paracetamol, the recommended acute treatment for migraine attacks during pregnancy, has been associated, on long-term use, with adverse neurodevelopmental outcomes [14], attention deficit hyperactivity disorder [15, 16], and hyperkinetic disorders [15] in children. Furthermore, a meta-analysis recently confirmed the association between the prenatal paracetamol exposure and the increased risk of child asthma [17]. In another meta-analysis which compared triptan-exposed women with the healthy controls during pregnancy, a significant increase in the rates of spontaneous abortions was found, while no increased risk of fetal malformations or prematurity was detected [18].

Notwithstanding the high prevalence of headache in women with childbearing potential [19], hence candidate to drug consumption during pregnancy, evidence about the safety of headache medication use during gestation is still limited. It is worth noting that most patients are not aware of the multifaceted risks due to migraine during pregnancy mentioned above: risks directly linked to the disease, risks due to its inappropriate treatment, and risks associated with headache medications [20]. In a Norwegian cross-sectional Internet-based survey [21], the majority of pregnant women and new mothers with migraine reported the intake of symptomatic medications during pregnancy, with a decreased use of triptans and nonsteroidal anti-inflammatory drugs (NSAIDs) in favor of paracetamol compared to the pregestational age, yet less than a third considered their headache to be optimally treated. Many women were concerned about whether it was safe to continue drug treatment during pregnancy and sought information about their medications [21]. Data about headache presentation and its treatment in pregnancy have never been collected in the Italian population. In this scenario, we aimed to investigate headache before and/or during pregnancy in a cohort of women, focusing on its pharmacological treatment, in terms of attitude on drug use, need of information on medications, and perception of the possible risks to the fetus deriving from drug consumption.

## Methods

We performed a retrospective cohort study with cross-sectional collection of data by convenience sampling of treatment-seeking individuals. From July 12, 2017, to September 2, 2017, women (≥ 18 years old) either in the last period of pregnancy (i.e., while they were admitted for planned delivery) or who have delivered in the previous 7 days were screened at the Maternal and Child Department, Careggi University Hospital, for enrolment in the study. Women who reported a diagnosis of secondary headaches, according to the *International Classification of Headache Disorders 3rd edition* (ICHD-3) [22], were not considered eligible to the study. Patients who reported having experienced headache before and/or during pregnancy were asked for filling a self-administered questionnaire, translated and adapted from Amundsen et al. [21]. The questionnaire was constituted by seven main sections: personal information, pregnancy information, headache history, headache presentation and medications before and during pregnancy, attitude toward use of headache medications before and during pregnancy, perception of fetal risk of drugs and substances during pregnancy, and research of information on headache medications before and during pregnancy (Table 1). We adapted and translated in Italian the Amundsen’s questionnaire (Supplementary Materials) in order to administer it to Italian-speaking participants.

**Table 1:**
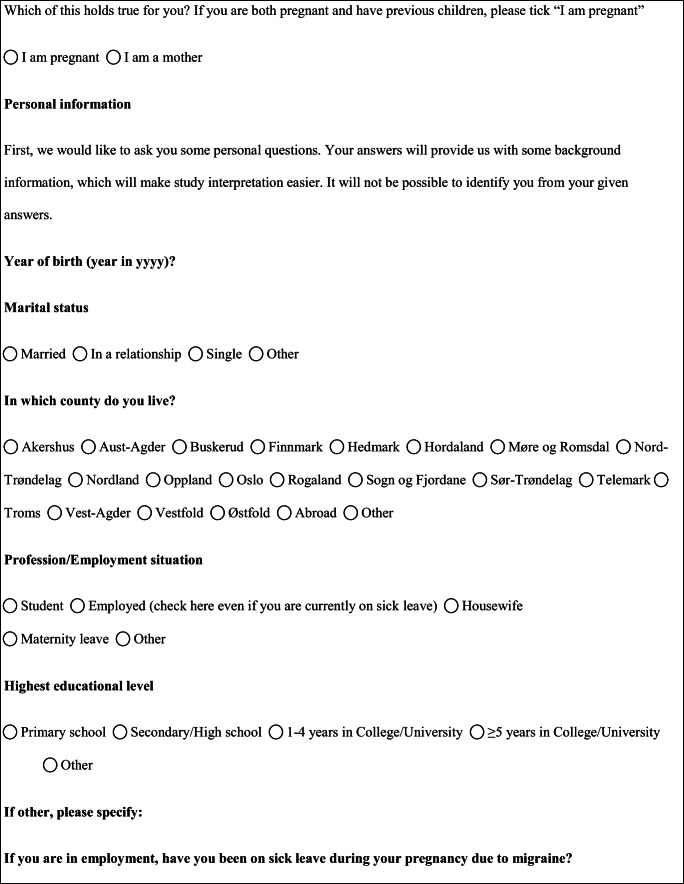

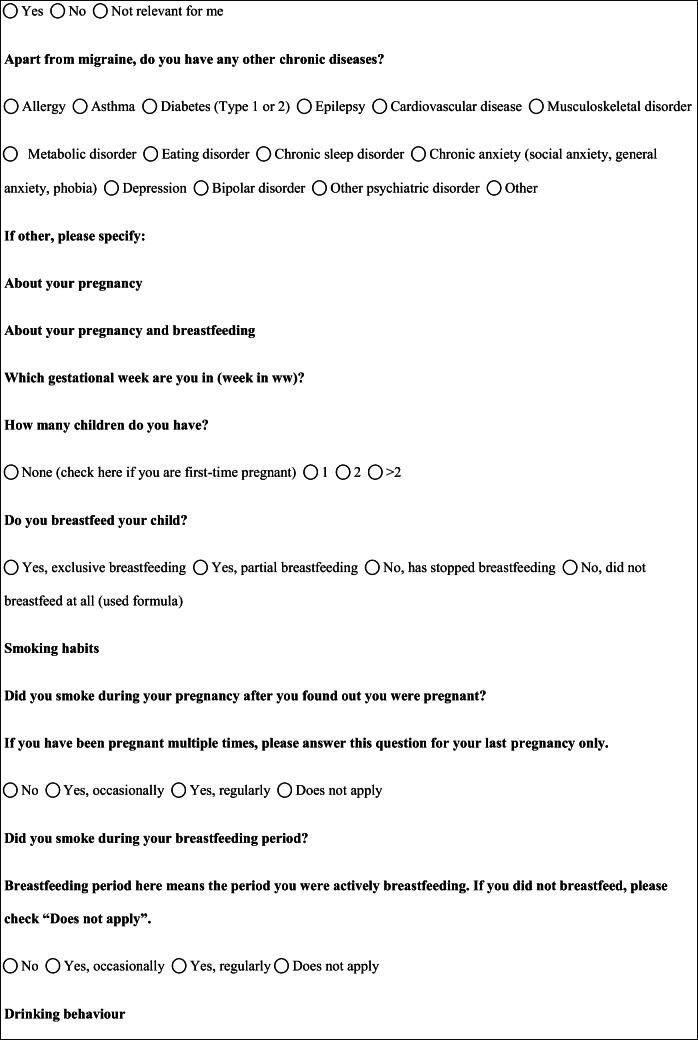

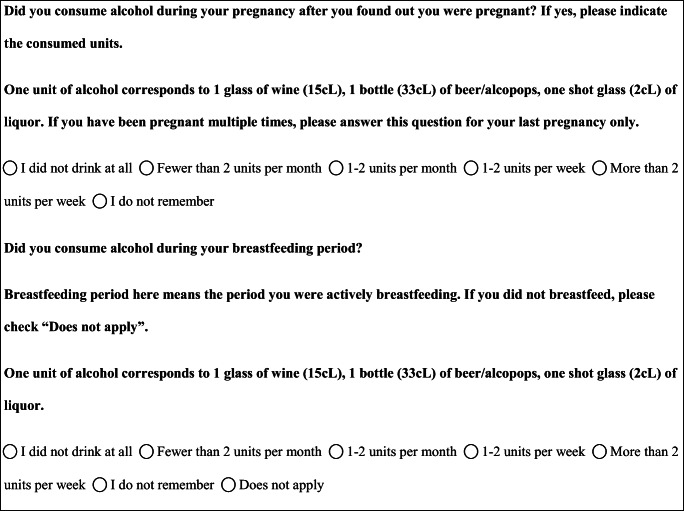

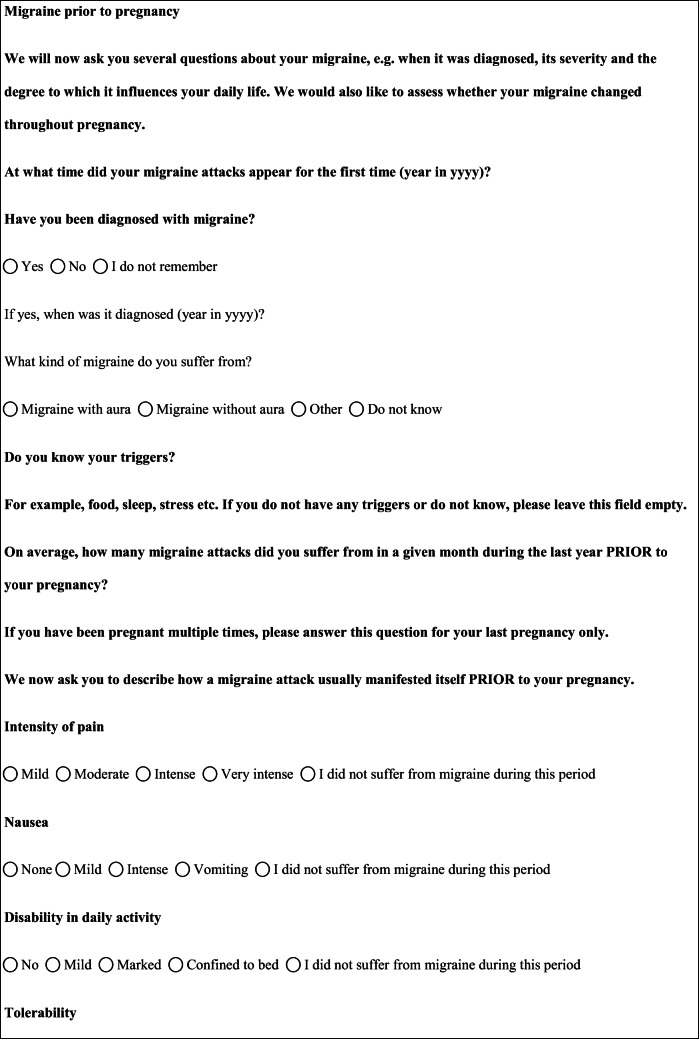

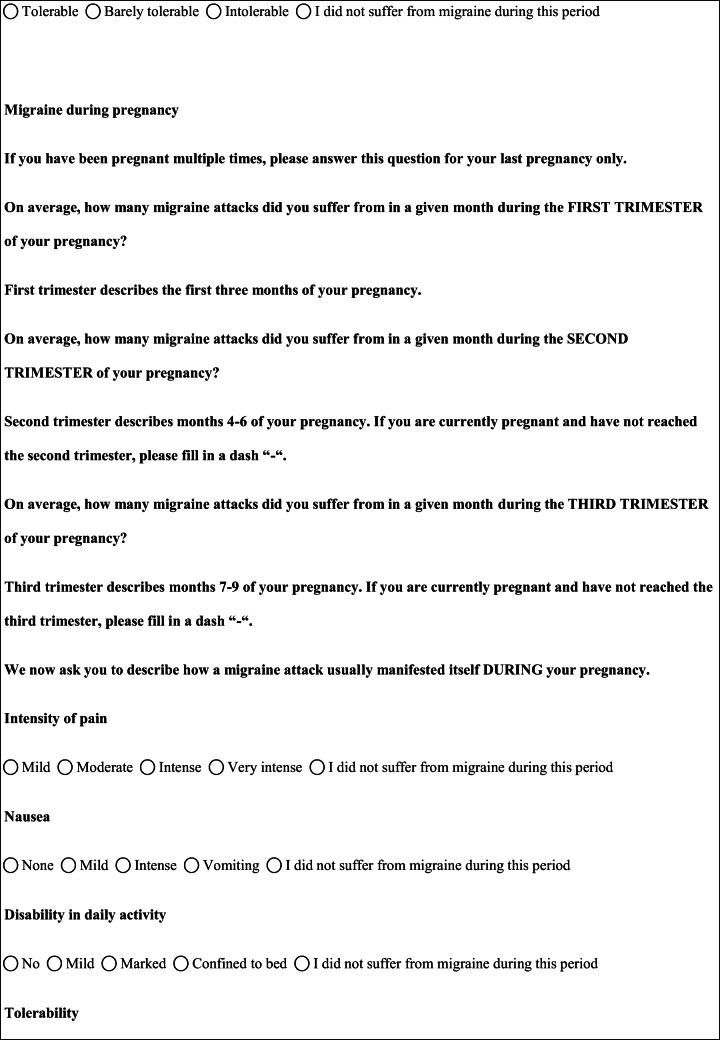

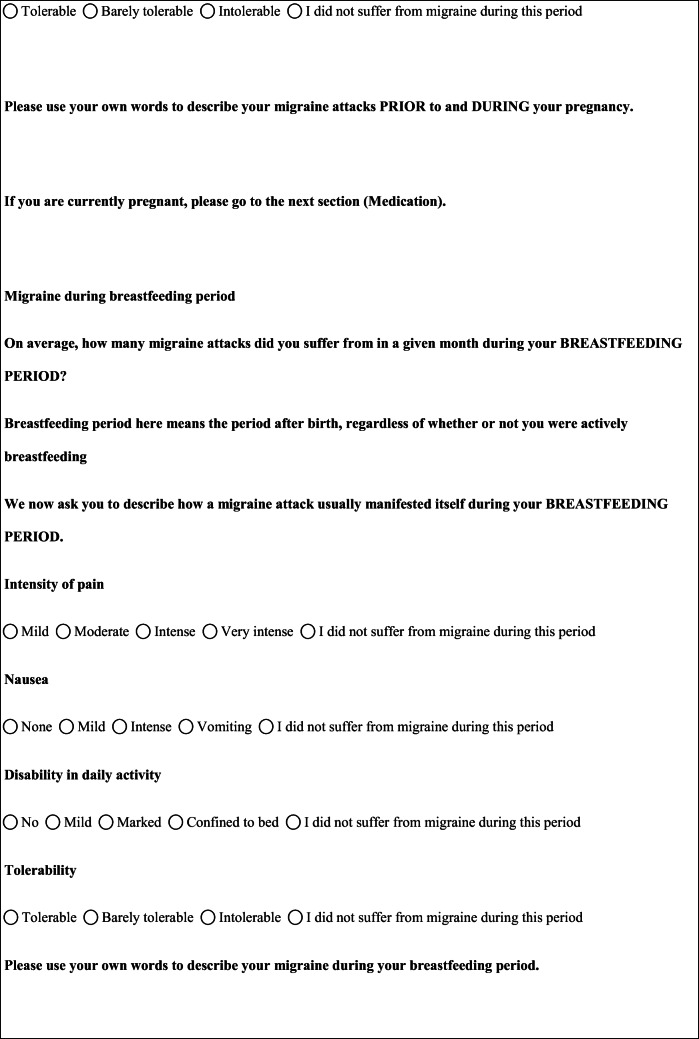

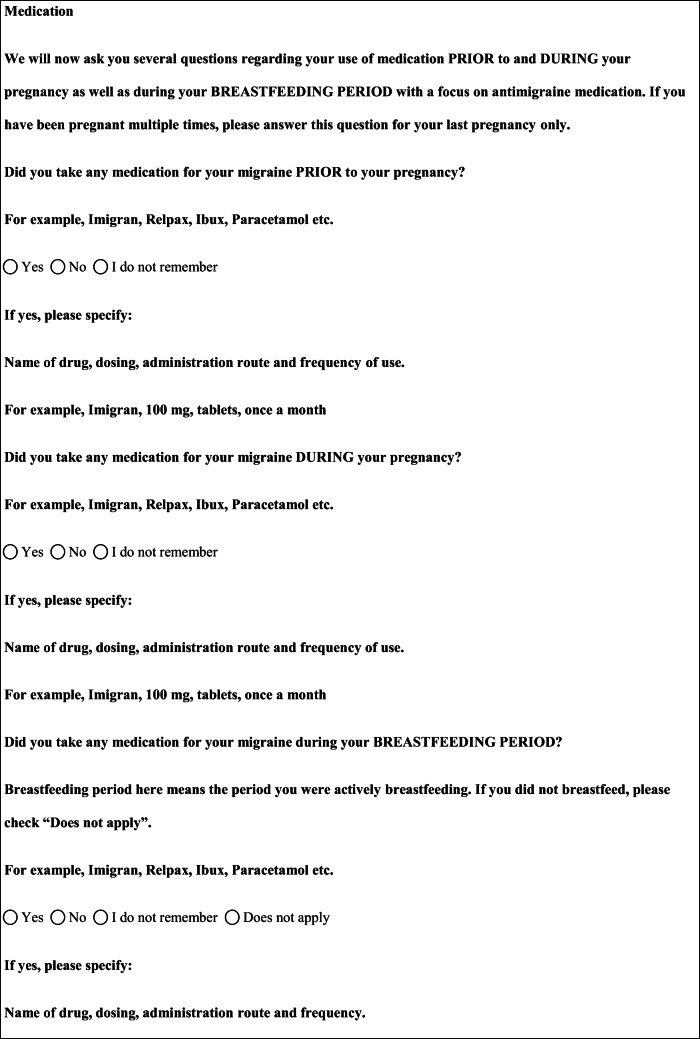

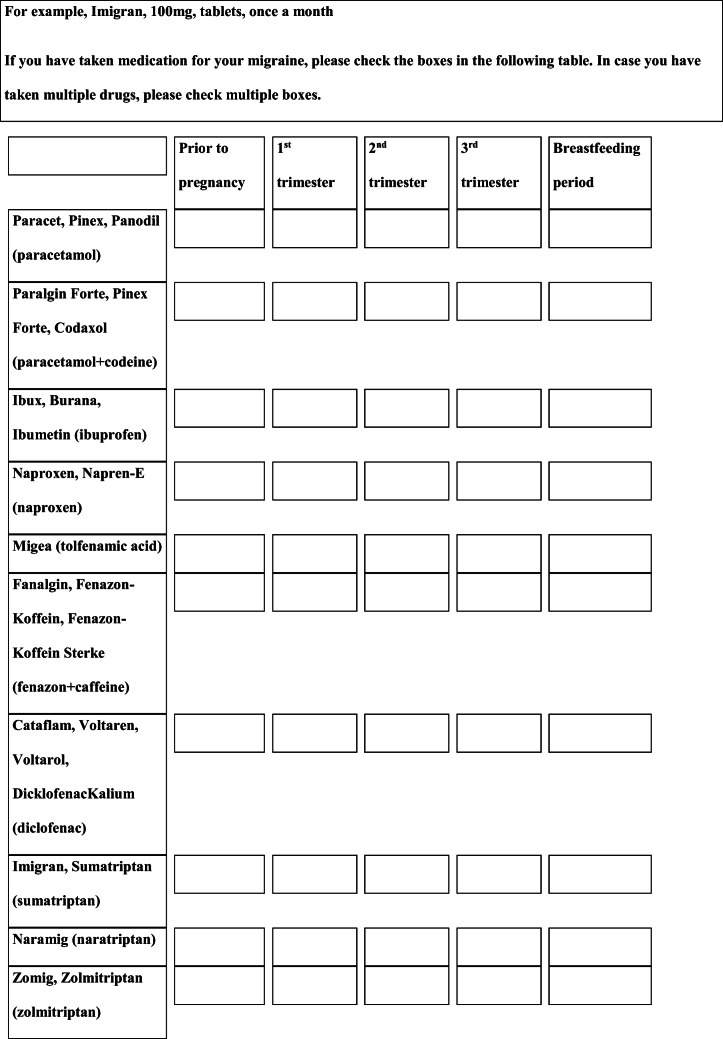

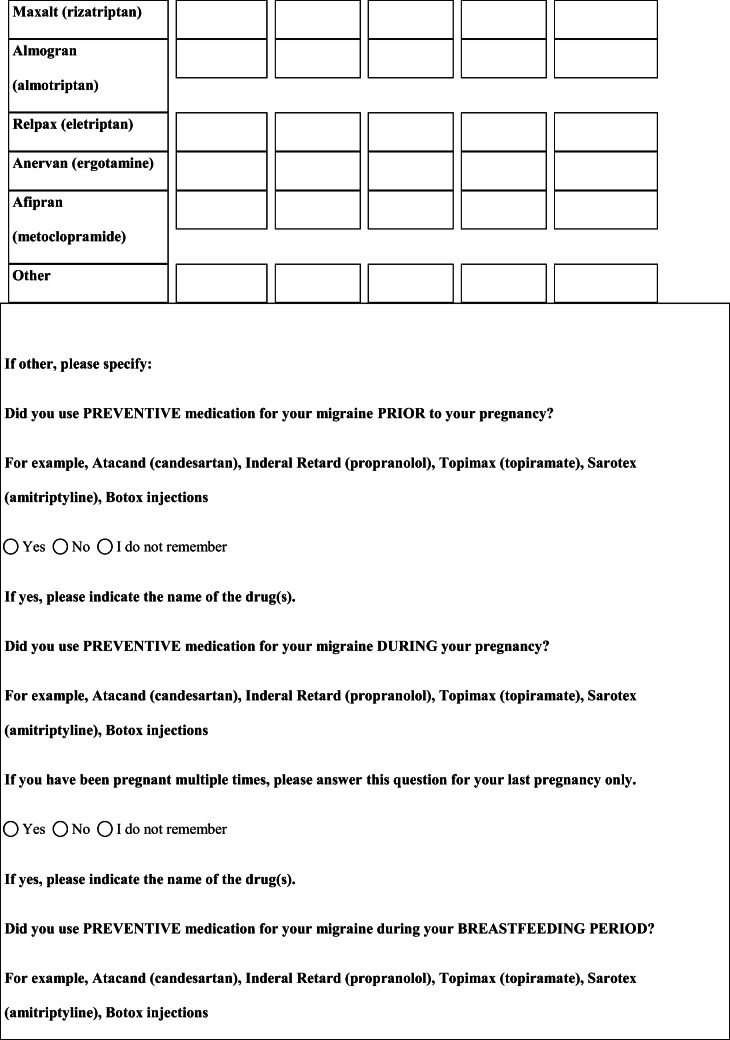

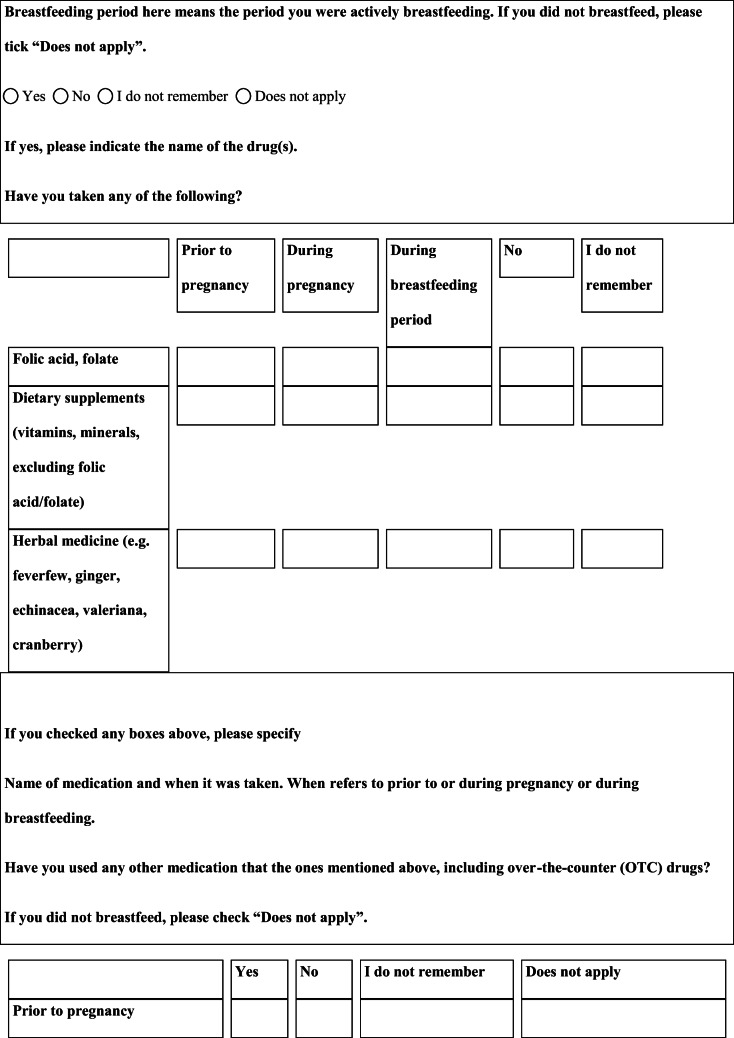

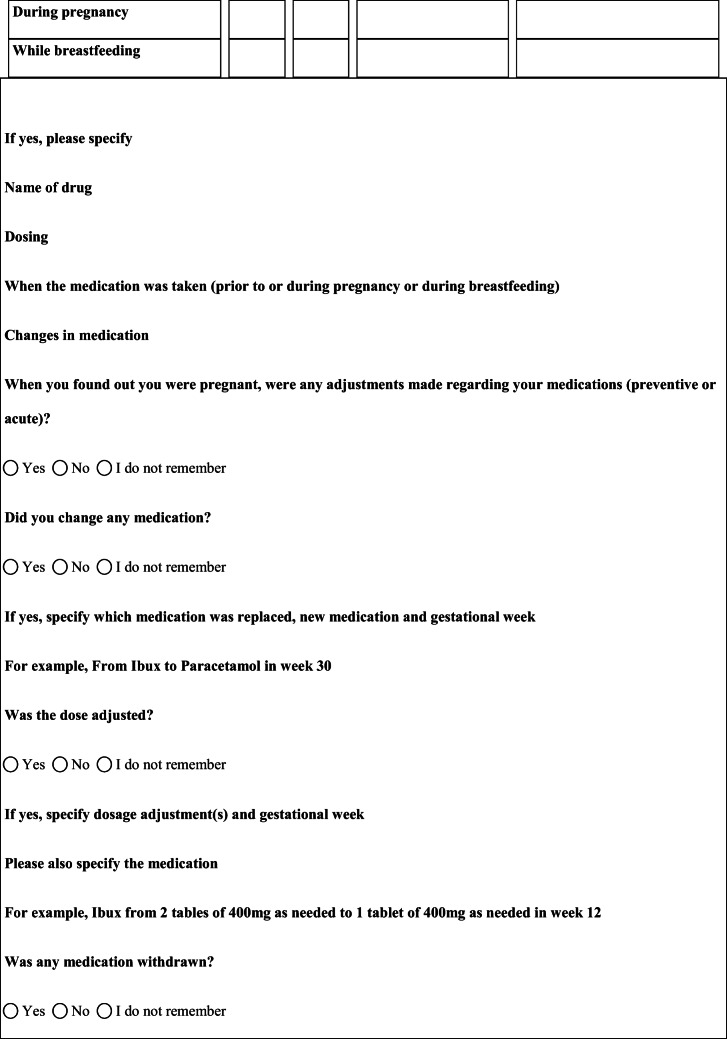

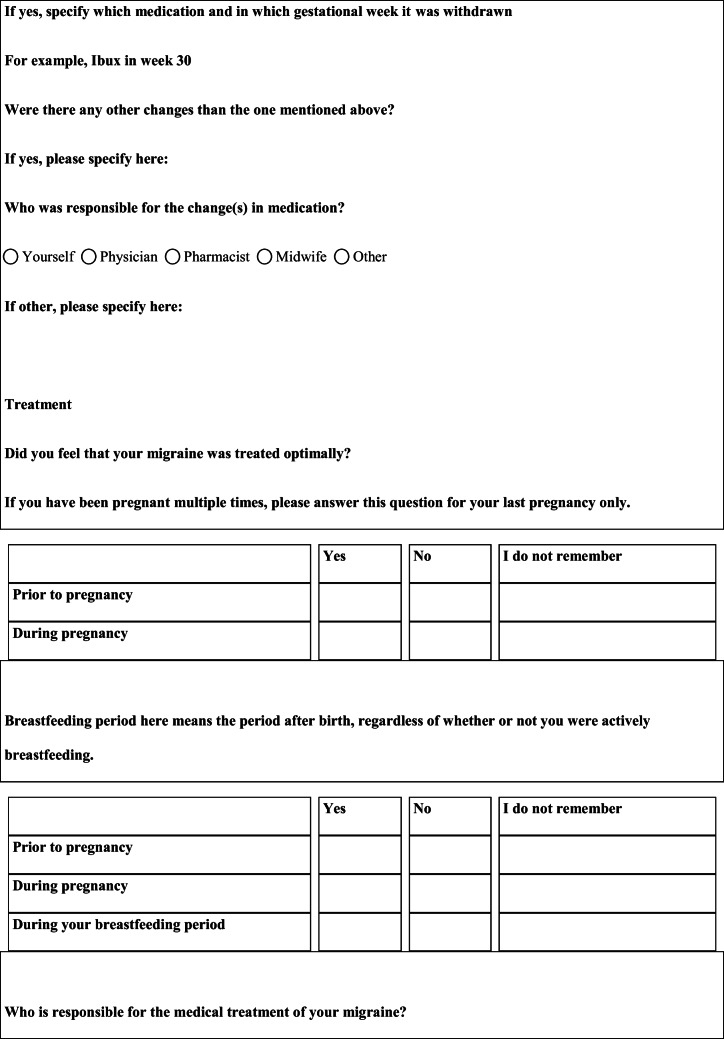

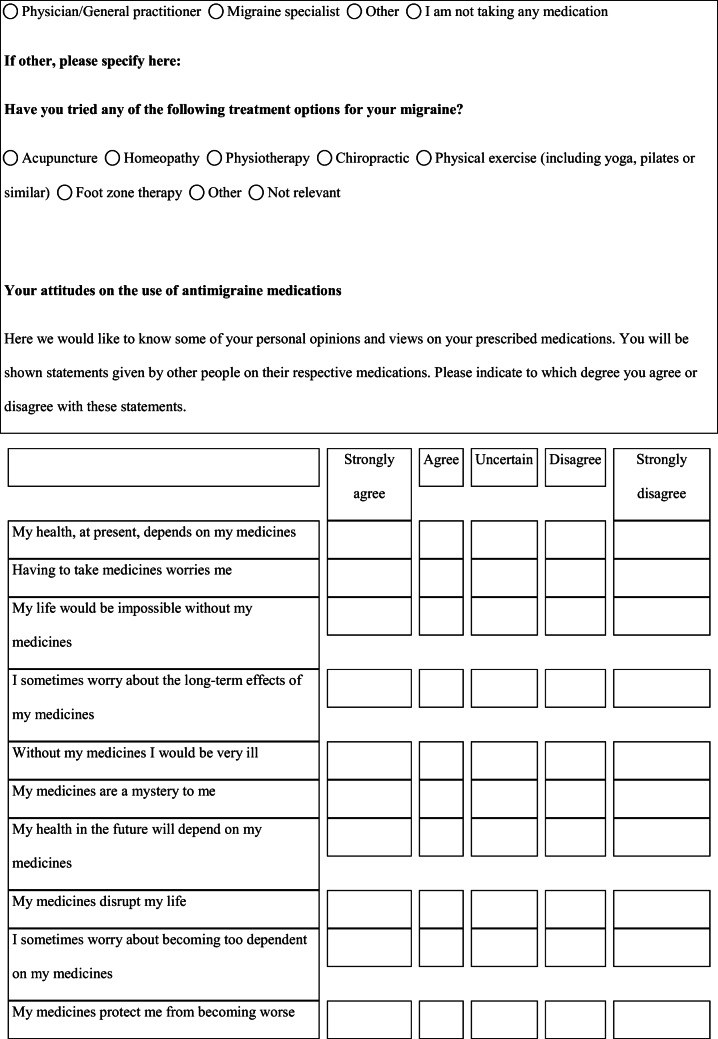

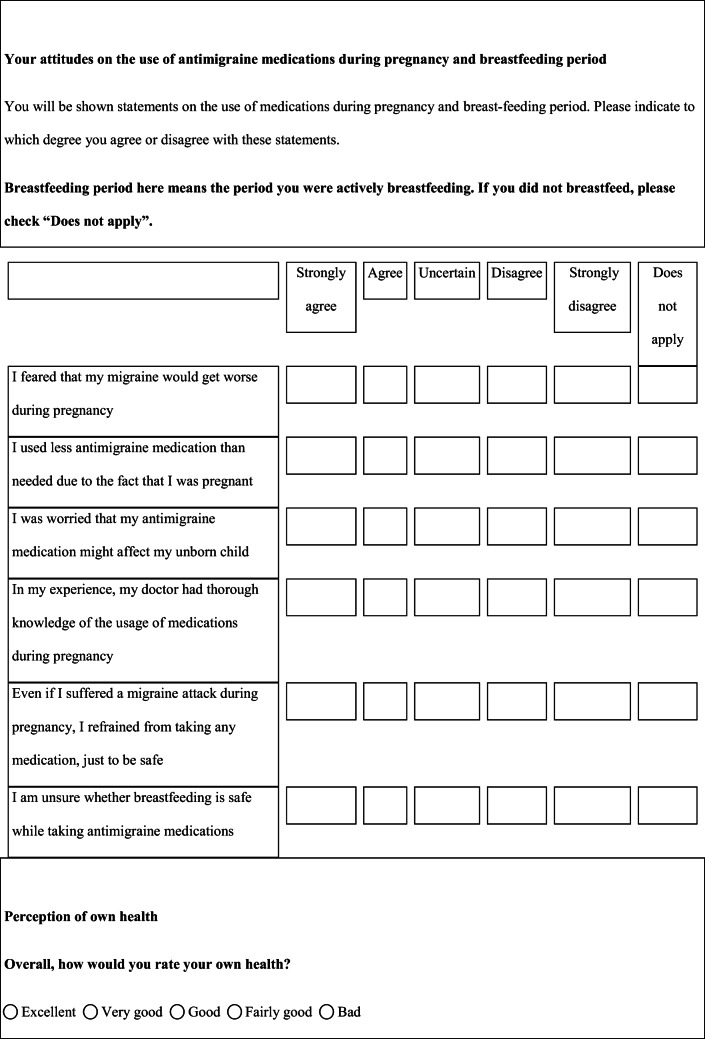

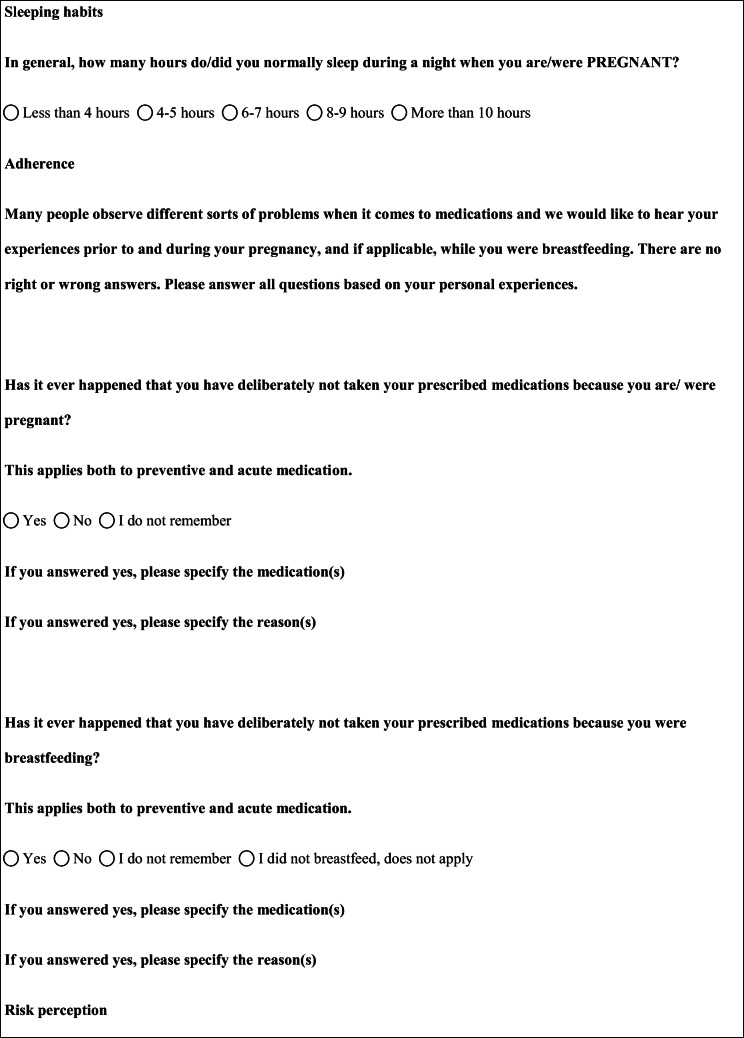

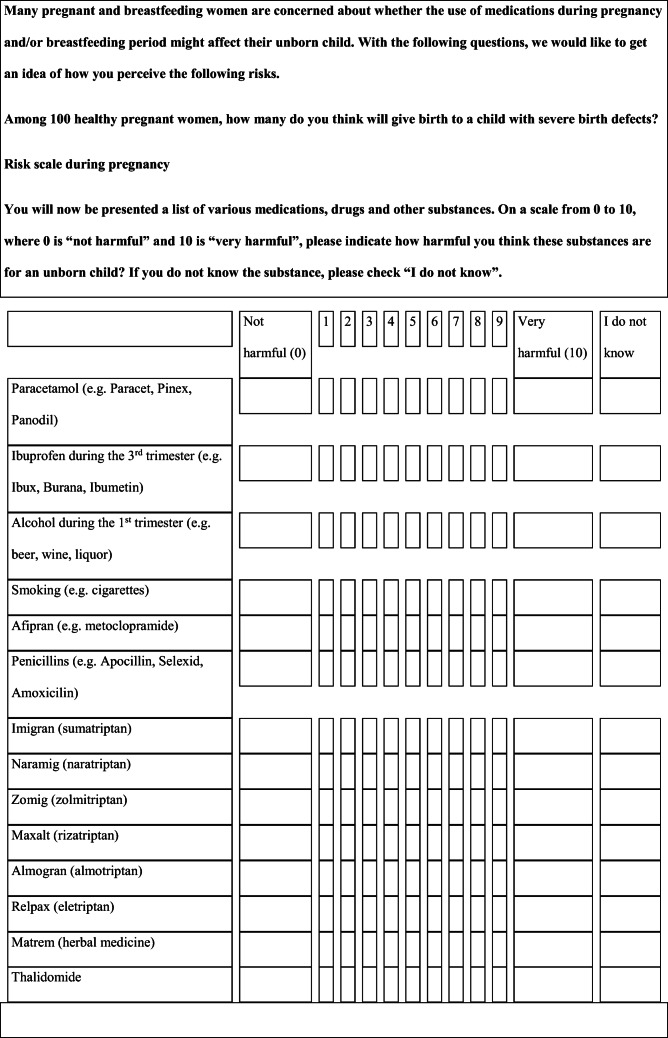

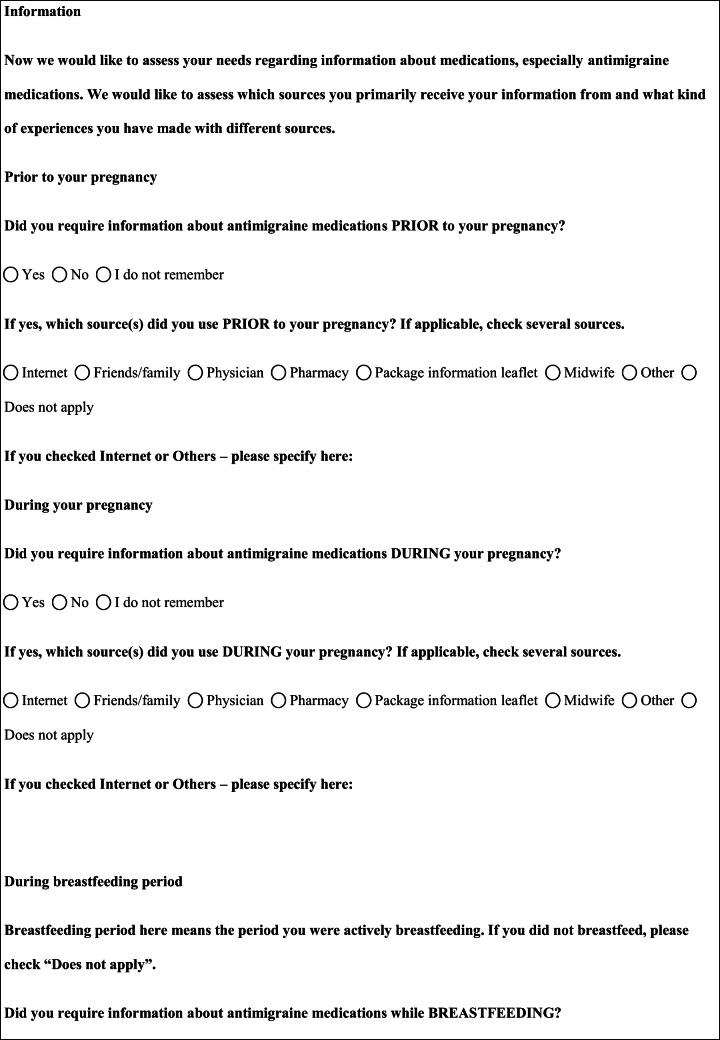

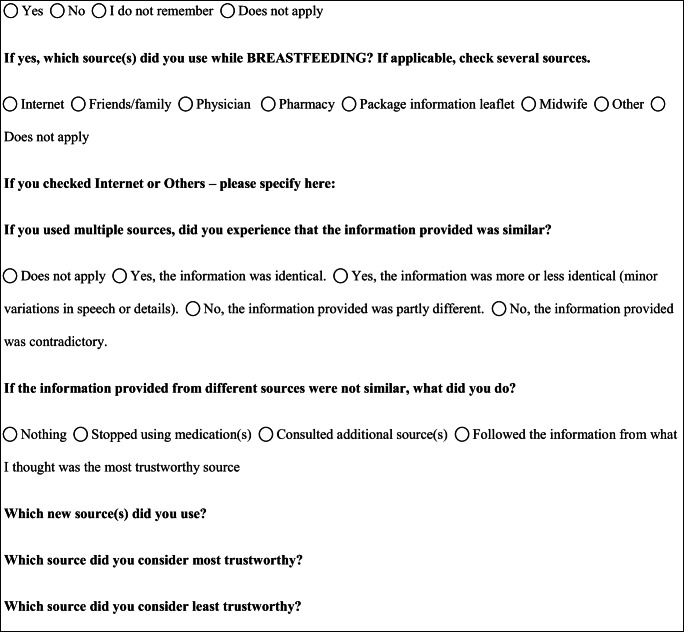
Original questionnaire from Amundsen et al. [21]

According to the descriptive aim of the study, no formal sample size calculation has been performed. The answers reported by the patients in the paper version of the questionnaire were transferred to an electronic database and analyzed by descriptive statistics (e.g., proportions and percentages for quantitative and categorical variables; mean ± SD or median and interquartile range (IQR) for continuous variables). The analysis was performed using Prism, 8.3.1 (GraphPad Software, San Diego, CA, USA). The participants were asked to express their opinion about headache medicine and substance consumption in pregnancy, in terms of perception of risk to the fetus, on a risk scale (from 0 [“not harmful”] to 10 [“very harmful”]). For analysis purposes, values ranging from 0 to 3 were considered as “low risk,” values from 4 to 6 were considered as “intermediate risk,” and values from 7 to 10 were considered as “high risk.” Original data are available on request from the authors.

## Results

### Study population

One hundred thirty-nine (51%) out of 271 screened women referred a history of headache, prior to and/or during pregnancy. One hundred women (37% of 271) signed the informed consent and participated in the study. Results are reported with detailed information about numbers and percentages of participants who gave answers. Numbers and percentages of patients that did not answer to each question, as they are low and can be easily inferred knowing numbers and percentages of participants who gave answers, are not detailed in the text unless they are relevant to the discussion.

### Personal information

Personal information of the study population included age, marital status, status of employment, highest level of education, sick leave during pregnancy due to headache, and chronic illnesses. This information was categorized and is reported in Table 2.

**Table 2:** Characteristics of the study population

Characteristics	Total *n* = 100, *n* (% of *n*)
Age	
≤ 30 years	11 (11)
31–35 years	42 (42)
≥ 36 years	47 (47)
Marital status	
Married/in relationship	97 (97)
Single/other	3 (3)
Status of employment	
Employed	71 (71)
Student/housewife/other	29 (29)
Level of education	
Academic degree	60 (60)
High school diploma	34 (34)
Middle school diploma	5 (5)
No answer	1 (1)
Sick leave	
Yes	7 (7)
No	82 (82)
Not applicable	11 (11)
Chronic illnesses	
None	70 (70)
Neuropsychiatric^a^	6 (6)
Cardiovascular^b^	6 (6)
Other^c^	20 (20)

^a^Epilepsy (3), depression (3), bipolar disorder (1), and insomnia (2)

^b^Cardiovascular disease (3) and metabolic disorder (3)

^c^Allergy (10), asthma (1), hypothyroidism (3), celiac disease (2), ulcerative colitis (1), anemia (1), autoimmune urticaria (1), and endometriosis (1)

### Pregnancy information

At the time of completion of the questionnaire, 77 participants (77%) referred they had delivered in the previous 7 days, and 22 (22%) referred they were pregnant (gestational age 37.3 ± 4.2 weeks). Fifty-four women (54%) reported to have already a child, 21 (21%) two children, and 10 (10%) more than two children. Fourteen women (14%) referred they were at their first pregnancy.

**Fig. 1:**
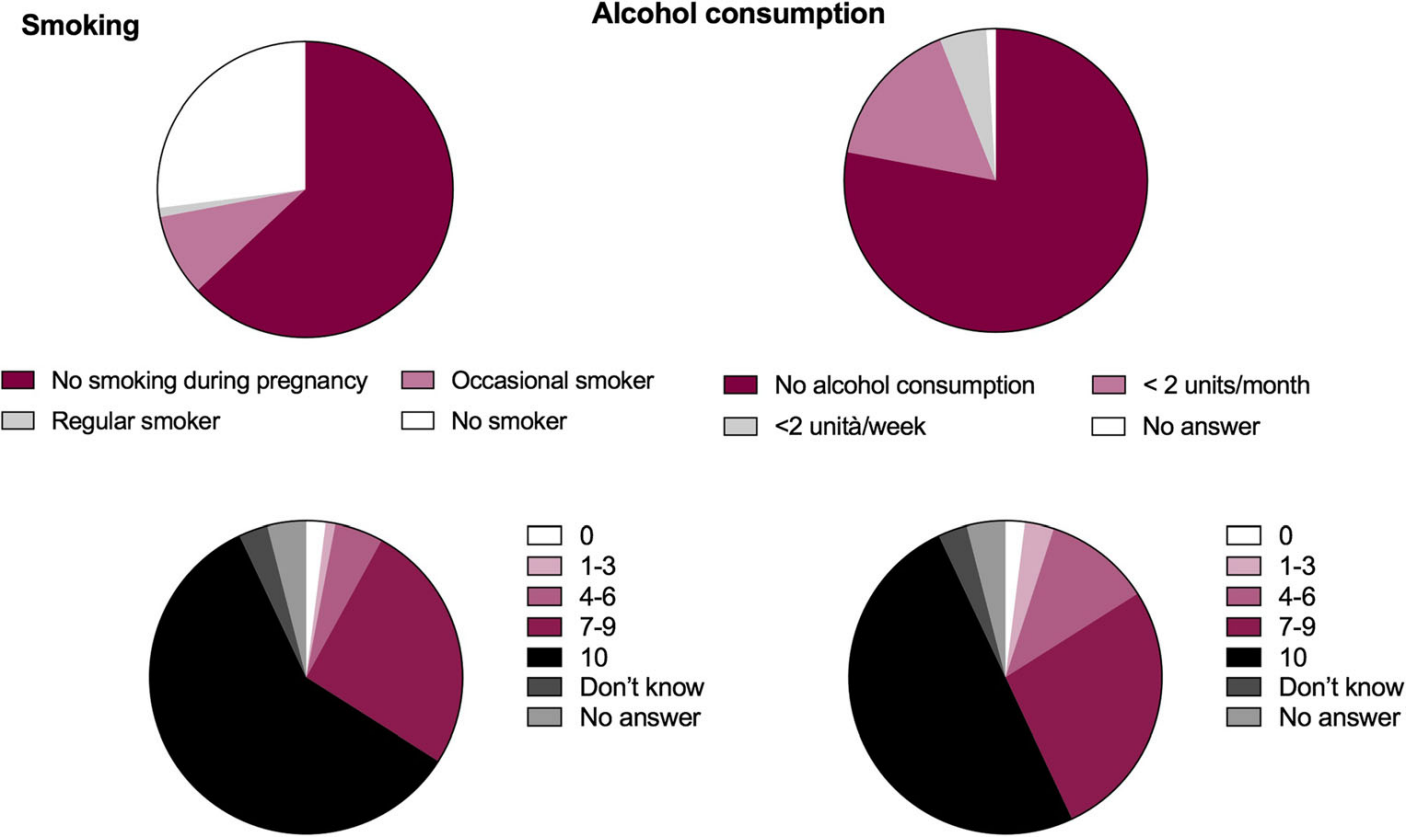
Smoking habit and alcohol consumption during pregnancy and, below, relative risk attributed to these behaviors. The perception of risk to the fetus was attributed using a numeric rating scale (NRS) ranging from 0 (“not harmful”) to 10 (“very harmful”)

Ten participants (10%) referred they had smoked during pregnancy, occasionally (9 participants) or regularly (1 participant). Sixty-three women (63%) reported they did not have smoked during gestation, and 27 (27%) reported they were not smokers even before pregnancy (Fig. 1).

In terms of alcohol use during pregnancy, 78 participants (78%) reported no alcohol consumption, while 21 (21%) reported alcohol use (Fig. 1).

### Headache history

Headache onset was reported to be, on average, at the age of 17.3 ± 6.3 by 90 women (90%); 10 women did not refer any information about that. Eighty-seven women (87%) referred headache attacks in the latest year before pregnancy. Twenty women reported they had received a headache diagnosis before pregnancy, including migraine (*n* = 8 migraine without aura, *n* = 7 migraine with aura), tension-type headache (*n* = 3), neck pain (*n* = 1), or other (*n* = 1). Headache diagnosis was done by a general practitioner (*n* = 6), a headache specialist (*n* = 6), or another physician (*n* = 3). Two women received the diagnosis by two different physicians (headache center specialist and general practitioner/other practitioner), while 3 did not specify the physician who performed the diagnosis.

### Headache presentation and medications before and during pregnancy

In the 87 women who referred headaches in the year before pregnancy, the median monthly frequency of headache attacks was 2 (IQR, ± 2.25); 4 women (4%) referred no headaches, while 9 (9%) did not report this information.

Sixty-two women (62%) reported headache attacks in the first trimester of gestation (2 ± 3.0), 44 (44%) in the second trimester (2 ± 1.5), and 43 (43%) in the third trimester (2 ± 1.75). Four women did not give any information about the number of headache crises during pregnancy. As a whole, 71 women referred headache attacks in at least one trimester of their pregnancy. The characteristics of headache attacks before and during pregnancy are described in Table 3.

**Table 3:** Characteristics of headache attacks

	Before pregnancy (total *n* = 87), *n* (% of *n*)	During pregnancy (total *n* = 71), *n* (% of *n*)
Intensity		
Mild	14 (16)	18 (25)
Moderate	36 (41)	37 (52)
Intense	21 (24)	11 (16)
Very intense	16 (19)	5 (7)
Nausea		
None	58 (67)	37 (52)
Mild	19 (22)	20 (28)
Intense	6 (7)	10 (14)
Vomiting	4 (4)	4 (6)
Daily disability		
No	37 (42)	29 (41)
Mild	24 (28)	26 (37)
Severe	17 (20)	13 (18)
Confined to bed	9 (10)	3 (4)
Tolerability		
Tolerable	37 (43)	36 (51)
Barely tolerable	29 (34)	27 (38)
Intolerable	20 (23)	8 (11)

**Fig. 2:**
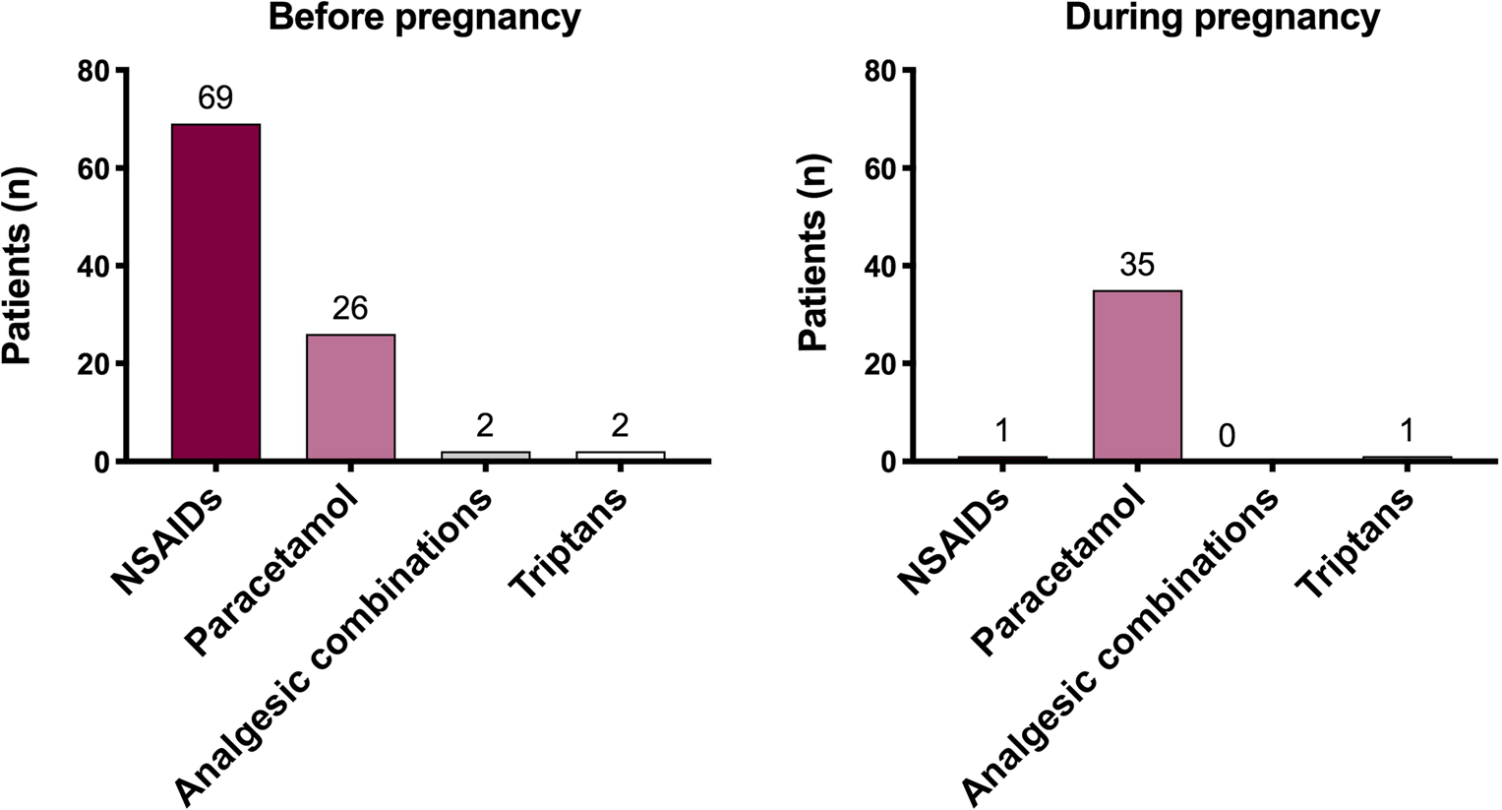
Class of drugs consumed before and during pregnancy to treat headache

Among the 87 patients who referred headache before pregnancy, 76 (88%) used drugs to treat the attacks, 9 (10%) did not use symptomatic medications, and 2 (2%) did not remember this information. The 76 women who treated headache attacks used NSAIDs (*n* = 52, 68%), paracetamol (*n* = 25, 33%), analgesic combinations (*n* = 2, 3%), and triptans (*n* = 2, 3%); each participant could indicate the use of more than one pharmacological class to treat the attacks (Fig. 2). Among the 71 women who referred headache attacks in at least one trimester of pregnancy, 37 (52%) used drugs to treat the attacks, 32 (45%) did not use symptomatic medications, and 2 (3%) did not remember this information. The 37 women who treated their headaches used paracetamol (*n* = 35, 95%), NSAIDs (*n* = 2 5%), and triptans (*n* = 1, 3%) (Fig. 2).

Just 6 women out of 87 (7%) reported the use of preventive therapy before pregnancy (i.e., 3 used magnesium supplementation, 1 reported homeopathy, and 3 did not specify the treatment) and 8 out of 71 (11%) during pregnancy (e.g., 4 reported magnesium supplementation, 2 reported iron supplementation, and 2 did not specify the treatment). Before pregnancy, 62 women out of 87 (71%) felt that their migraine was treated optimally, 18 (21%) felt that their migraine was not optimally treated, and 7 (8%) did not remember or did not answer the question. During pregnancy, 56 women out of 71 (79%) felt that their migraine was treated optimally, 4 (6%) felt that their migraine was not optimally treated, and 11 women (15%) did not remember or did not answer the question. Twenty-two women among the 87 (25%) with headaches in the year before pregnancy reported that some adjustments have been made regarding their headache medications (preventive or acute) when they found out they were pregnant. Seventeen women specified that the adjustment consisted in the substitution of NSAIDs (i.e., 8 used ibuprofen, 4 nimesulide, 4 ketoprofen, and 1 aspirin) with paracetamol. Thirty-four women out of 87 (39%) reported the withdrawal of headache medications during pregnancy; 24 reported the withdrawal of a NSAID (i.e., 13 ibuprofen, 6 nimesulide, and 5 ketoprofen), 3 reported the withdrawal of 2 NSAIDs (i.e., 1 ibuprofen and ketoprofen and 2 ibuprofen and nimesulide), 1 reported the interruption of indomethacin-caffeine-prochlorperazine, and another reported the interruption of eletriptan. Fifty-one women out of 87 (59%) reported a responsible for the abovementioned changes in medications, while 36 (41%) did not answer this question. Twenty-eight women reported they autonomously decided these changes, and 20 attributed the responsibility to the practitioner and a woman to the pharmacist. Two women reported more than one responsible for the changes (i.e., a woman the practitioner and the midwife, while another woman answered she decided together with the pharmacist). Seventy-nine women out of 87 (91%) indicated a responsible for the medical treatment of headache before and during pregnancy. As responsible for the medical treatment of headache, 33 women reported the general practitioner, a woman reported the neurologist, 64 women reported the headache center specialist, 25 women answered “other,” and 19 women answered “I am not taking any medication.” Among the 25 women who answered “other,” 15 reported they were responsible of their own treatment, 2 reported the pharmacist was the responsible, 2 referred the complementary and alternative medicine physicians, a woman referred the gynecologist, and a woman referred the toxicologist. Four women referred more than one responsible for the medical treatment of headache.

### Attitude toward use of headache medications

The attitude of the participants toward use of headache medications before and during pregnancy is reported in Tables 4 and 5, respectively. The questions about the attitude toward drug use before pregnancy were answered by those participants who suffered headache before pregnancy (*n* = 87). The questions about the attitude toward drug use in pregnancy were answered by those participants who suffered headache during that period (*n* = 71).

**Table 4:** Attitude of the study population toward the use of headache drugs before pregnancy

	Attitude toward use of headache medications before pregnancy (total *n* = 87), *n* (% of *n*)									
	My health, at present, depends on my medicines	My medicines are a mystery to me	Having to take drugs worries me	My health in the future will depend on my medicines	My life would be impossible without my medicines	My medicines disrupt my life	I sometimes worry about the long-term effects of my medicines	I sometimes worry about becoming too dependent on my medicines	Without my medicines, I would be very ill	My medicines protect me from becoming worse
Strongly agree	3 (3)	2 (2.5)	8 (9)	0 (0)	1 (1)	1 (1)	6 (7)	5 (6)	0 (0)	8 (9)
Agree	15 (17)	2 (2.5)	23 (27)	6 (7)	7 (8)	3 (3)	30 (34)	7 (8)	5 (6)	38 (44)
Uncertain	8 (9)	8 (9)	14 (16)	17 (20)	11 (13)	2 (2)	14 (16)	6 (7)	8 (9)	12 (14)
Disagree	18 (21)	35 (40)	25 (29)	24 (28)	22 (25)	33 (38)	22 (25)	30 (34)	27 (31)	13 (15)
Strongly disagree	38 (44)	35 (40)	15 (17)	37 (42)	43 (49)	44 (51)	11 (13)	37 (43)	44 (51)	13 (15)
No answer	5 (6)	5 (6)	2 (2)	3 (3)	3 (4)	4 (5)	4 (5)	2 (2)	3 (3)	3 (3)

**Table 5:** Attitude of the study population toward the use of drugs for treating headache during pregnancy

	Attitude toward the use of headache medications during pregnancy (total *n* = 71), *n* (% of *n*)				
	I feared that my headache would get worse during pregnancy	I used less headache medication than needed due to the fact that I was pregnant	I was worried that my headache medication might affect my unborn child	In my experience, my doctor had thorough knowledge of the medication usage during pregnancy	Although I suffered headache in pregnancy, I refrained from using any medication, just to be safe
Strongly agree	3 (4)	31 (44)	18 (25)	17 (24)	21 (30)
Agree	18 (25.5)	22 (31)	25 (35)	36 (51)	11 (15)
Uncertain	12 (17)	2 (3)	5 (7)	3 (4)	1 (1)
Disagree	18 (25.5)	5 (7)	11 (16)	4 (6)	22 (31)
Strongly disagree	15 (21)	7 (10)	8 (11)	5 (7)	12 (17)
No answer	5 (7)	4 (5)	4 (6)	6 (8)	4 (6)

Twenty-five subjects reported they had decided not to use symptomatic drugs during pregnancy (10 women specified they did not use paracetamol, 6 ibuprofen, 1 nimesulide, 2 ketoprofen, and 1 rizatriptan; 5 did not give drug details).

### Perception of fetal risk of various drugs and substances during pregnancy

Answering the question “Among 100 healthy pregnant women, how many do you think will give birth to a child with severe birth defects?,” 63 women (63%) estimated a mean value of 4.4 children born with severe birth defects, while 37 (37%) did not report the data (33 did not answer, and 4 answered they did not know).

**Fig. 3:**
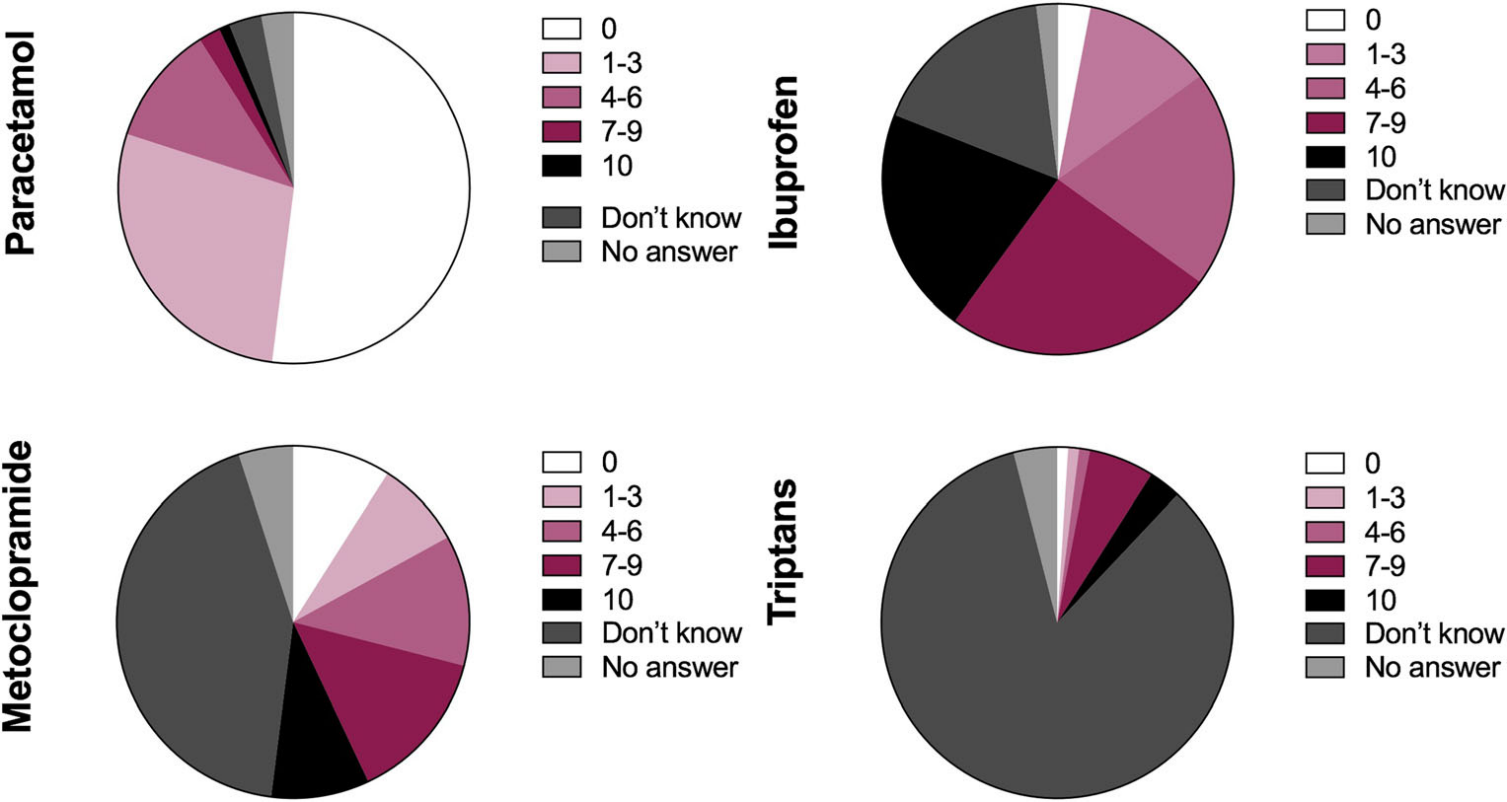
Risk attributed by participants to headache medicines (i.e., paracetamol, ibuprofen, metoclopramide, and triptans). The perception of risk to the fetus was attributed using a numeric rating scale (NRS) ranging from 0 (“not harmful”) to 10 (“very harmful”)

Eighty participants (80%) attributed a low risk (risk class “0–3”) to the use of paracetamol, 11 (11%) an intermediate risk (risk class “4–6”), and 3 (3%) a high risk (risk class “7–10”), while 6 did not report a value (3 did not answer, and 3 answered they did not know) (Fig. 3, paracetamol risk). Fifteen women (15%) attributed low risk to the use of ibuprofen in the third trimester of gestation, 20 (20%) intermediate risk, and 46 (46%) high risk, while 19 did not report this information (17 did not answer, and 2 answered they did not know) (Fig. 3, ibuprofen risk). Seventeen women (17%) attributed low risk to the use of metoclopramide, 12 (12%) intermediate risk, and 23 (23%) high risk, while 48 women did not report this information (43 did not answer, and 5 answered they did not know) (Fig. 3, metoclopramide risk). The largest part of the women did not know triptans and, therefore, was not able to attribute a risk class to these medicines (*n* = 84, 84%). Just 12 participants (12%) attributed a risk class to triptans; two (2%) attributed them low risk, 2 (2%) intermediate risk, and 8 (8%) high risk (Fig. 3, triptan risk). Smoking was given low risk by 3 women (3%), intermediate risk by 5 (5%), and high risk by 85 (85%). Seven women (7%) did not attribute any class risk to tobacco smoke (4 did not answer to the question, and 3 answered they did not know) (Fig. 1). Alcohol intake during the first trimester of pregnancy was associated to low risk by 5 women (5%), intermediate risk by 11 (11%), and high risk by 77 (77%). Seven women (7%) did not attribute to alcohol any class risk (4 women did not answer, and 3 women answered they did not know) (Fig. 1).

### Research of information on headache medications before and during pregnancy

**Fig. 4:**
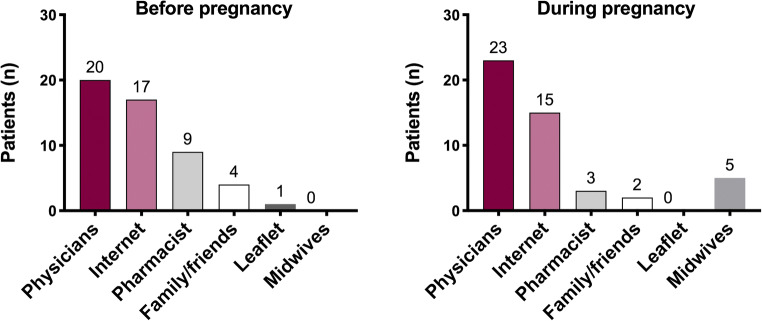
Source of information about headache drugs before and during pregnancy

Thirty-one women (36%) of the 87 suffering from headache before pregnancy reported to have searched information about headache medications prior to the pregnancy and the information sources were represented by physicians (*n* = 20), the Internet (*n* = 17), pharmacy (*n* = 9), friends/family (*n* = 4), and leaflet (*n* = 1). Fifty-one participants (58%) reported no information search, while 5 (6%) did not remember or did not report this data (Fig. 4). Information need was similar during pregnancy, with 31 women (31 out of 71, 44%) who referred to have required information to physicians (*n* = 23), the Internet (*n* = 15), pharmacy (*n* = 3), friends/family (*n* = 2), and midwives (*n* = 5), and 37 women (52%) without information needs. Three participants (4%) did not remember or did not report this data (Fig. 4). Thirteen women out of 31 (42%) who searched for information during pregnancy reported they have consulted multiple sources. Five of them declared that the information provided was identical among the different sources, 2 reported that the information provided was mostly identical, and 5 referred that the information was more or less identical. Just one woman reported that the information provided was contradictory and she had followed the information from the trust worthiest source according to her opinion (i.e., the physician).

## Discussion

A general, initial comment to our results is that according to the high rate (51%) of women suffering from headache among those attending the Maternal and Child Department, this setting should be considered optimal to enroll patients in future studies collecting data about headache and migraine. Going into depth on results, among the participants, 80% had not received a headache diagnosis before pregnancy and this explains the prevalent use of nonspecific headache medicines. However, some data from this study may suggest that there were many women with migraine among them. First of all, the young age at the onset of a headache that lasts over time with a frequency of 3.7 ± 5.7 is strongly suggestive of migraine. Second, 84% of participants reported moderate to severe headaches before pregnancy which were more than 50% of the time barely tolerable or intolerable and associated with disability and in 34% associated with nausea (including vomiting in 4%) (Table 3). All these features strongly suggest migraine. Third, the number of reported headache attacks decreased during pregnancy, with a marked reduction in the transition from the first to the second trimester (62% against 44%) and then remained stable until the end of pregnancy (43%). This tendency in the frequency of headache attacks is typical of the clinical course of migraine during pregnancy, as demonstrated in several previous studies [23].

The results show important changes in behavior and therapeutic habits once pregnancy has begun. In fact, before pregnancy, almost all patients (88%) used to treat headache attacks with drugs, but this percentage decreases significantly during pregnancy when almost half of women (45%) did not take symptomatic drugs. The reduction in symptomatic intake was also accompanied by a change in the type of class of drugs used. Compared to the previous year, during pregnancy, the proportion of participants who used NSAIDs was reduced from 68 to 5%, while that of those who used paracetamol increased from 33 to 95% (Fig. 2). The 2 patients who used combinations of analgesics suspended them in pregnancy, as did one of the 2 women who treated their attacks with triptans.

From the analysis of the data regarding the attitude of the patients toward the use of symptomatic drugs before the pregnancy, it emerges a concern about the safety of drugs and, more importantly, the willingness to avoid a possible dependence from them. The majority replied that they did not feel that neither their current health nor their future health depends on drugs (65% and 70%, respectively), and they did not worry about having to take drugs (46%) but about their long-term effects (38%) or the idea of becoming dependent on them (77%). In addition, participants report that the drugs protect them from getting worse (55%), but without thinking that without them, they would be very ill (82%) or that their life would be impossible (74%). Despite this, the attitude toward taking drugs in our sample has changed during pregnancy, reflecting a protective interest in the child as demonstrated by the fact that 25% avoided taking drugs. However, even among those who used symptomatic drugs, most women reported using fewer medicines than they needed because they were pregnant (75%) and because they feared they could harm their child (60%), although believing that their doctor had a thorough knowledge of these medicines (75%). Of interest, excluding a minority that was uncertain about the course that the headache would have had (17%), half of the patients feared a worsening during pregnancy while the other half was not afraid. This last finding is justified by the fact that 80% of women suffered from headaches of which they did not know the causes and for which they had not received a diagnosis.

Importantly, both women who have discontinued the use of symptomatic drugs and those who have used them have shown that they have obtained adequate information about their safety during pregnancy. Correctly, 80% of women attributed to paracetamol a low risk and 11% an intermediate risk, while with regard to the use of ibuprofen during the third trimester, 46% indicated a high risk and 20% an intermediate risk. Regarding the use of metoclopramide, women showed less knowledge about its safety, and in fact among those who expressed an opinion, 33% attributed a low risk, 23% an intermediate risk, while 44% a high risk. Notably, not only triptans are only marginally used, but the majority of patients are not able to attribute a risk to their use, corroborating the hypothesis that these drugs still remain a niche treatment. On the other side, women showed a good knowledge of the effects of some voluptuous habits during pregnancy, attributing a high risk to alcohol intake during the first trimester (77%) and smoking throughout the pregnancy (85%). The final test of the information on pregnancy status comes from the question of what the incidence of newborns with congenital defects in 100 healthy pregnant women was. A percentage of sixty-three of women who answered this question gave an average estimate of 3.4 which fits perfectly in the 3–5% estimate reported in the literature [24, 25].

Regarding the use of preventive therapy, it was low both before and during pregnancy (7% and 11% of respondents, respectively) and limited to magnesium supplementation. Magnesium, like most nutraceuticals, has a low level of recommendation according to international migraine treatment guidelines [26]. However, it is commonly used as a self-prescribed anxiolytic agent and in the case of nonspecific headaches and, together with the fact that 80% of the participants suffered from an ever before diagnosed headache, this could explain why magnesium was the most frequently used preventive treatment. Notably, some patients report the use of iron supplementation for headache prevention; also, this result may suggest a scarce knowledge about headache and the need for more education.

Interesting evidence emerges analyzing the need for information on headache drugs, with a change in the attitude before and during pregnancy, although less marked than for the intake of drugs. The percentage of women who searched for information during pregnancy was slightly higher than that in the pre-pregnancy period (44% vs. 36%), and the proportion of those that did not look for them earlier decreased slightly during pregnancy (58% vs. 52%). The main sources of information were doctors and the Internet, with a small prevalence of doctors during pregnancy and the Internet if before pregnancy. However, 42% of women who sought information during pregnancy reported having consulted several sources (e.g., pharmacists, friends, family, midwives) and that the answers were identical or roughly identical.

### Limitations of the study

This study has some methodological limitations. First, data collection was performed using a self-administrated questionnaire and, although synthetic and with a linear flow of questions, it is possible that there were errors in interpretation and compilation. Second, the retrospective nature of our analysis is per se a drawback and there is the possibility of recall bias, although the timespan is relatively short. Moreover, for each question in the questionnaire, there were a certain number of women who did not provide an answer. Third, this study was conducted in one institution and, consequently, the findings cannot be generalized to all Italian regions.

## Conclusions

There are several noteworthy findings emerging from this study. First, one pregnant woman out of two reported to have suffered from headache, suggesting that maternal departments may be taken into consideration to enroll patients in studies collecting data about headache and migraine. Second, in our cohort, the large majority of patients reporting headache have never received a diagnosis, suggesting the need to both increase the patients’ awareness for searching medical attention and ameliorate the diagnostic process. Finally, it is worth noting that pregnancy determines relevant changes in how women treat their headache and, in some extent, how they search for information on drug safety, mostly due to perception of fetal risk of medicines. Accordingly, healthcare providers, in order to optimize headache treatment during pregnancy, should be aware they have to face particular needs of pregnant women with headache, namely pregnancy-oriented education and prescription changes.

## Electronic supplementary material

ESM 1 (DOC 116 kb)

## Data Availability

The dataset generated and analyzed during the current study is available from the corresponding author on reasonable request.

## Abbreviations

NRS: Numeric rating scale
NSAIDs: Nonsteroidal anti-inflammatory drugs
