# Supplementary material for: Medicines for headache before and during pregnancy: a retrospective cohort study (ATENA study)
Source: Neurol Sci. 2020 Sep 23;42(5):1895–921. doi: 10.1007/s10072-020-04702-0 (PMC8043957; doi:10.1007/s10072-020-04702-0)
Supplement: Supplementary file 1 — (DOC 116 kb) [file 10072_2020_4702_MOESM1_ESM.doc]

**IMPIEGO DI FARMACI PER IL TRATTAMENTO DELLA CEFALEA ED INFORMAZIONI SUL LORO USO IN GRAVIDANZA: UNO STUDIO DI COORTE RETROSPETTIVO (STUDIO ATENA)**

**QUESTIONARIO PER LA PAZIENTE**

Codice Identificativo: …………………………….

**Se Lei è attualmente incinta**, Le viene chiesto di rispondere relativamente alla gravidanza in corso.

**Se Lei è stata incinta nei precedenti 6 mesi**, Le viene chiesto di rispondere solo ed esclusivamente relativamente alla gravidanza appena trascorsa.

**INFORMAZIONI PERSONALI** (Non sarà possibile identificarLa dalle risposte da Lei fornite)

**Anno di nascita?** ………………………………

**Stato civile** ⃝ Sposata ⃝ Relazione ⃝ Single ⃝ Altro

**Professione o Situazione lavorativa**

⃝ Studentessa ⃝ Occupata (faccia la crocetta qui, anche se attualmente Lei è in congedo malattia/maternità) ⃝ Casalinga ⃝ Congedo maternità ⃝ Altro

**Livello di istruzione più elevato** ⃝ Scuola primaria ⃝ Media inferiore ⃝ Media superiore ⃝ Università

**Se è occupata, ha dovuto prendere giorni di congedo malattia a causa del mal di testa durante la gravidanza?**

⃝ Sì ⃝ No ⃝ Non rilevante per me

**A parte il mal di testa, Lei soffre di qualche altra malattia cronica?**

⃝ Allergie ⃝ Asma ⃝ Diabete (Tipo 1 o 2) ⃝ Epilessia ⃝ Malattia cardiovascolare ⃝ Disordine muscoloscheletrico ⃝ Disordine metabolico ⃝ Disordine alimentare ⃝ Insonnia ⃝ Disturbo d’ansia cronico (ansia sociale, disturbo d’ansia generalizzato, fobie) ⃝ Depressione ⃝ Disturbo bipolare ⃝ Altro disturbo psichiatrico ⃝ Altro (specificare):…………………………………………………………….

**INFORMAZIONI SULLA GRAVIDANZA**

**In quale settimana di gestazione è? (rispondere se Lei è attualmente incinta)** …………………………………

**Quanti figli ha?** ⃝ Nessuno (in caso di prima gravidanza) ⃝ 1 ⃝ 2 ⃝ >2

**Ha fumato durante la gravidanza, dopo aver scoperto di essere incinta?**

⃝ No ⃝ Sì, occasionalmente ⃝ Sì, regolarmente ⃝ Non applicabile (in caso di paziente non fumatrice)

**Ha consumato alcol in gravidanza, dopo aver scoperto di essere incinta? Se sì, indichi le unità consumate**

Un’unità alcolica corrisponde a un bicchiere di vino (15cL) o ad una bottiglietta (33cL) di birra/bevanda alcolica o ad un bicchierino (2cL) di liquore.

⃝ Non ho bevuto alcol ⃝ Meno di 2 unità al mese ⃝ 1-2 unità al mese ⃝ 1-2 unità a settimana ⃝ Più di 2 unità settimana ⃝ Non ricordo

**CEFALEA A che età sono comparsi per la prima volta attacchi di mal di testa?** …………………………..

**Le è stata mai diagnosticata da un medico (es. medico di famiglia, etc) una forma specifica di mal di testa?**  ⃝ Sì ⃝ No ⃝ Non ricordo

**Se ha risposto Sì alla domanda precedente, quale tipo di mal di testa le è stato diagnosticato e da quale professionista?**  ⃝ Emicrania senz’aura ⃝ Emicrania con aura ⃝ Cefalea di tipo tensivo ⃝ Altro (specificare:………………………..) ⃝ Medico di famiglia ⃝ Neurologo ⃝ Medico di un Centro Cefalee ⃝ Altro (specificare:………………………..)

**CEFALEA PRIMA DELLA GRAVIDANZA**

**In media, di quanti attacchi di mal di testa al mese ha sofferto nell’ultimo anno prima della gravidanza?** ………

**Come si manifestava in genere un attacco di mal di testa prima della gravidanza?**

- **Intensità del dolore**

⃝ Lieve ⃝ Moderata ⃝ Intensa ⃝ Molto intensa ⃝ Non ho sofferto di emicrania prima della gravidanza

- **Nausea**

⃝ No ⃝ Lieve ⃝ Intensa ⃝ Vomito ⃝ Non ho sofferto di emicrania prima della gravidanza

- **Disabilità nelle attività quotidiane**

⃝ No ⃝ Lieve ⃝ Marcata ⃝ Confinata a letto ⃝ Non ho sofferto di emicrania prima della gravidanza

- **Tollerabilità**

⃝ Tollerabile ⃝ Appena tollerabile ⃝ Intollerabile ⃝ Non ho sofferto di emicrania prima della gravidanza

**CEFALEA DURANTE LA GRAVIDANZA**

**In media, di quanti attacchi di mal di testa al mese ha sofferto nel PRIMO TRIMESTRE di gravidanza?** …………….

**In media, di quanti attacchi di mal di testa al mese ha sofferto nel SECONDO TRIMESTRE di gravidanza?** .....….. (Se non ha ancora raggiunto il secondo trimestre di gravidanza, riempia con il simbolo “-“)

**In media, di quanti attacchi di mal di testa al mese ha sofferto nel TERZO TRIMESTRE di gravidanza?** ....………… (Se non ha ancora raggiunto il terzo trimestre di gravidanza, riempia con il simbolo “-“)

**Come si è manifestato in genere un attacco di mal di testa durante la gravidanza?**

- **Intensità del dolore**

⃝ Lieve ⃝ Moderata ⃝ Intensa ⃝ Molto intensa ⃝ Non ho sofferto di emicrania durante la gravidanza

- **Nausea**

⃝ No ⃝ Lieve ⃝ Intensa ⃝ Vomito ⃝ Non ho sofferto di emicrania durante la gravidanza

- **Disabilità nelle attività quotidiane**

⃝ No ⃝ Lieve ⃝ Marcata ⃝ Confinata a letto ⃝ Non ho sofferto di emicrania durante la gravidanza

- **Tollerabilità**

⃝ Tollerabile ⃝ Appena tollerabile ⃝ Intollerabile ⃝ Non ho sofferto di emicrania durante la gravidanza

**FARMACI PER LA CURA DELLA CEFALEA**

**Ha assunto farmaci per trattare gli attacchi di mal di testa prima della gravidanza?**

⃝ Sì ⃝ No ⃝ Non ricordo

Se sì, specificare nome, dose, via di somministrazione e frequenza d’uso (ad es. Brufen, 600 mg, cp, 1 volta al mese; MAX 3 FARMACI): ……………………………………………………………...............................................

**Ha assunto farmaci per trattare gli attacchi di mal di testa durante la gravidanza?**

⃝ Sì ⃝ No ⃝ Non ricordo

Se sì, specificare nome, dose, via di somministrazione e frequenza d’uso (ad es. Brufen, 600 mg, cp, 1 volta al mese; MAX 3 FARMACI): ……………………………………………………………...............................................

**Ha assunto farmaci o integratori per la prevenzione del di mal di testa prima della gravidanza?**

⃝ Sì ⃝ No ⃝ Non ricordo/non so

Se sì, indicare il nome dell’ultimo farmaco o integratore usato (ad es. Laroxyl, Mag2, ecc): …………………

**Ha assunto farmaci o integratori per la prevenzione del di mal di testa durante la gravidanza?**

⃝ Sì ⃝ No ⃝ Non ricordo/non so

Se sì, indicare il nome dell’ultimo farmaco o integratore usato (ad es. Laroxyl, Mag2, ecc): …………………

**Quando ha scoperto di essere in stato interessante, ci sono state sostituzioni nei farmaci per il mal di testa (sintomatici o di prevenzione)?**

⃝ Sì ⃝ No ⃝ Non ricordo

Se sì, specificare (ad es. da Brufen 600 mg a Tachipirina 1000 mg alla 30^a^ settimana): ……………………………

**Ha interrotto l’assunzione di qualche farmaco/i per il mal di testa?**

⃝ Sì ⃝ No ⃝ Non ricordo

Se sì, quale farmaco/i e in quale/i settimana/e gestazionale? ………………………………………………………………

**Chi è stato responsabile del/i cambiamento/i nei farmaci per il mal di testa?**

⃝ Io stessa ⃝ Medico ⃝ Farmacista ⃝ Ostetrica ⃝ Altro (specificare): ……………………………….

**Pensa che il Suo mal di testa sia stata trattato in modo ottimale?**

**Prima della gravidanza** ⃝ Sì ⃝ No ⃝ Non ricordo **Durante la gravidanza**  ⃝ Sì ⃝ No ⃝ Non ricordo

**Chi è stato responsabile del trattamento della Suo mal di testa?**

⃝ Medico di famiglia ⃝ Neurologo ⃝ Medico del Centro Cefalee ⃝ Altro (specificare): …………………… ⃝ Non prendo farmaci

**ATTEGGIAMENTO SULL’USO DEI FARMACI**

|  | Concordo fortemente | Concordo | Sono incerta | Non sono d’accordo | Non sono affatto d’accordo |
| --- | --- | --- | --- | --- | --- |
| La mia salute, al momento, dipende dalle mie medicine |  |  |  |  |  |
| Dover prendere delle medicine mi preoccupa |  |  |  |  |  |
| La mia vita sarebbe impossibile senza le mie medicine |  |  |  |  |  |
| Talvolta mi preoccupo delle conseguenze a lungo termine derivanti dall’uso delle mie medicine |  |  |  |  |  |
| Senza le mie medicine sarei molto malata |  |  |  |  |  |
| Le mie medicine sono un mistero per me |  |  |  |  |  |
| La mia salute in furo dipenderà dalle mie medicine |  |  |  |  |  |
| Le mie medicine mi distruggono la vita |  |  |  |  |  |
| Talvolta mi preoccupo della possibilità di diventare troppo dipendente dalle mie medicine |  |  |  |  |  |
| Le mie medicine mi proteggono dalla possibilità di stare peggio |  |  |  |  |  |

**ATTEGGIAMENTO SULL’USO DEI FARMACI IN GRAVIDANZA (Rispondere in caso di attacchi di mal di testa in gravidanza)**

|  | Concordo fortemente | Concordo | Sono incerta | Non sono d’accordo | Non sono affatto d’accordo |
| --- | --- | --- | --- | --- | --- |
| Temevo che il mio mal di testa peggiorasse in gravidanza |  |  |  |  |  |
| Ho usato meno farmaci per il mal di testa del necessario, perché sono incinta |  |  |  |  |  |
| Sono preoccupata che i farmaci per il mal di testa possano recare danni al nascituro |  |  |  |  |  |
| Nella mia esperienza, il mio medico era pienamente a conoscenza dell’impiego dei farmaci in gravidanza |  |  |  |  |  |
| Anche se ho avuto mal di testa, per sicurezza non ho mai preso un farmaco per il mal di testa in gravidanza |  |  |  |  |  |

**È mai accaduto che Lei abbia per scelta non assunto i farmaci (sintomatici o di profilassi) prescritti per il mal di testa, perché incinta?**

⃝ Sì ( specificare il/i farmaco/i e la/e ragione/i:………………………………………………………………………………………………..) ⃝ No ⃝ Non ricordo

**PERCEZIONE DEL RISCHIO IN GRAVIDANZA**

**Su 100 donne incinta e sane, quante pensa che daranno alla luce un bambino con severi difetti alla nascita?**…

**Scala del rischio in gravidanza** In una scala da 0 a 10, dove 0 è “non dannoso” e 10 è “molto dannoso”, quanto pensa siano dannose le sostanze/farmaci sotto elencati per il nascituro?

|  | Non dannoso (0) | 1 | 2 | 3 | 4 | 5 | 6 | 7 | 8 | 9 | Molto dannoso (10) | Non so |
| --- | --- | --- | --- | --- | --- | --- | --- | --- | --- | --- | --- | --- |
| Paracetamolo (es. Tachipirina, Efferalgan) |  |  |  |  |  |  |  |  |  |  |  |  |
| Ibuprofene nel 3^o^ trimestre (es. Brufen, Moment) |  |  |  |  |  |  |  |  |  |  |  |  |
| Alcool nel 1^o^ trimestre |  |  |  |  |  |  |  |  |  |  |  |  |
| Fumo |  |  |  |  |  |  |  |  |  |  |  |  |
| Metoclopramide (es. Plasil) |  |  |  |  |  |  |  |  |  |  |  |  |
| Sumatriptan (Imigran) |  |  |  |  |  |  |  |  |  |  |  |  |
| Frovatriptan (Auradol, Rilamig) |  |  |  |  |  |  |  |  |  |  |  |  |
| Zolmitriptan (Zomig) |  |  |  |  |  |  |  |  |  |  |  |  |
| Rizatriptan (Rizaliv, Maxalt) |  |  |  |  |  |  |  |  |  |  |  |  |
| Almotriptan (Almogran) |  |  |  |  |  |  |  |  |  |  |  |  |
| Eletriptan (Relpax) |  |  |  |  |  |  |  |  |  |  |  |  |

**RICERCA DI INFORMAZIONI SULLA CEFALEA**

**Ha ricercato informazioni sui farmaci per il mal di testa?**

**Prima della gravidanza** ⃝ Sì ⃝ No ⃝ Non ricordo **Durante la gravidanza** ⃝ Sì ⃝ No ⃝ Non ricordo

**Se sì, quali fonti ha usato prima della gravidanza?**

⃝ Internet ⃝ Depliant ⃝ Amici/Famiglia ⃝ Farmacia ⃝ Ostetrica ⃝ Medico

**Se sì, quali fonti ha usato durante la gravidanza?**

⃝ Internet ⃝ Depliant ⃝ Amici/Famiglia ⃝ Farmacia ⃝ Ostetrica ⃝ Medico

**Se ha usato più fonti, ha verificato che l’informazione fornita fosse simile?**

⃝ Non applicabile ⃝ Sì, l’informazione era identica ⃝ Sì, l’informazione era più o meno identica ⃝ Sì, l’informazione era in buona parte identica ⃝ No, le informazioni erano contraddittorie

**Se le informazioni fornite da diverse fonti non erano simili, cosa ha fatto?**

⃝ Niente ⃝ Ho sospeso l’assunzione di farmaci ⃝ Ho consultato altre fonti (specificare): ………………….. ⃝ Ho seguito l’informazione fornita dalla fonte per me più fidata (specificare): ………………………………......
